# Ideal Nasal Angle Preferences Among Japanese People: A Prospective Observational Study on Aesthetic Perception

**DOI:** 10.1093/asjof/ojaf052

**Published:** 2025-06-24

**Authors:** Kohki Okumura, Takahiko Tamura, Yusuke Funakoshi, Hiroo Teranishi

## Abstract

**Background:**

The aesthetic ideal of the nose varies across nationalities and cultures, making it challenging to define an “ideal” nasal shape in rhinoplasty.

**Objectives:**

The authors of this study aimed to provide foundational data for generalizable aesthetic standards for nasal surgery in Japanese individuals.

**Methods:**

A randomly selected group of 783 Japanese adults was presented with digitally modified images depicting 12 nasolabial and nasofacial angles. Aesthetic evaluations were conducted using Google Forms or paper surveys. The images were categorized into 3 groups: “aesthetic,” “average,” and “not aesthetic.” Statistical analysis was conducted using JMP software. To minimize bias, the order in which the images were presented was randomized. The Steel–Dwass test was used to assess statistically significant differences between angles.

**Results:**

For men, a nasolabial angle of 95° and a nasofacial angle of 33° were rated as aesthetic, whereas for women, a nasolabial angle of 105° and a nasofacial angle of 30° were considered favorable. The results indicate a preference for sharper nasolabial angles in men and slightly obtuse angles in women. When calculating the ideal angles using the weighted average, the ideal nasolabial angle for men was 105° and the ideal nasofacial angle was 30°. For women, the ideal nasolabial and nasofacial angles were 105° and 30°, respectively.

**Conclusions:**

The authors of this study highlight sex-specific nasal preferences within the Japanese population and emphasize the importance of tailoring rhinoplasty procedures to demographic factors.

**Level of Evidence: 5 (Therapeutic):**

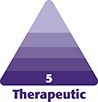

Aesthetic perceptions of the nose differ significantly among different nationalities and cultures, complicating the development of universal standards for rhinoplasty. In recent years, the demand for nasal cosmetic surgery has surged, driven by the increased emphasis on individualized aesthetic outcomes that reflect personal and cultural preferences. For Japanese individuals, achieving a harmonious nasal shape that aligns with facial features is especially important in cosmetic surgery; however, current standards are limited by insufficient data tailored specifically to Japanese anatomy and aesthetic ideals. This limitation underscores the clinical significance of establishing comprehensive population-specific criteria to guide rhinoplasty outcomes in Japanese patients.

Despite efforts to define the ideal nasal angles for Japanese individuals, previous studies have often been constrained by methodological limitations, such as small sample sizes, selection biases, and outdated datasets. For instance, Takahashi identified an optimal nasolabial angle of ∼118.32° in the Japanese population.^1^ However, such studies lack generalizability because of limited participant diversity and often focus on narrow demographic groups that may not accurately represent broader societal preferences.

Therefore, the purpose of this study was to identify culturally preferred nasolabial and nasofacial angles among Japanese adults using a large sample and digitally manipulated images created with 3-dimensional (3D) imaging technology. We hypothesized that the use of a highly representative sample and advanced imaging techniques would produce accurate and generalizable data that can guide culturally sensitive rhinoplasty practices in Japan.

## METHODS

### Study Design and Timeline

**Figure 1.**
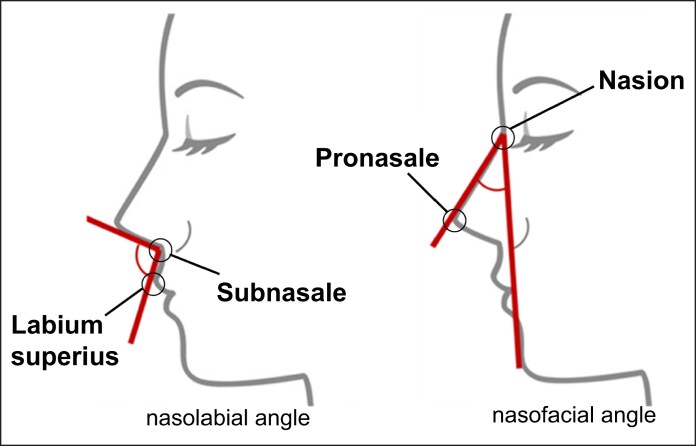
Representative illustrations of nasal angles analyzed in this study. The nasolabial angle is defined as the angle formed at the subnasale by the intersection of 2 straight lines: 1 tangent to the base of the nose (columella nasi) and the other tangent to the outer contour of the upper lip (labium superius). The nasofacial angle is defined as the angle formed between a line drawn from the nasion to the pronasale and a vertical line perpendicular to the Frankfurt horizontal plane. Reference points: nasion: the midpoint of the frontonasal suture at the root of the nose; pronasale: the most protruding point of the nasal tip; subnasale: the point where the nasal septum meets the upper lip; columella nasi: the tissue at the nasal base between the nostrils; labium superius: the most anterior point on the upper lip vermilion border.

This prospective observational cohort study was conducted from July to October 2024. The methodology followed the Strengthening the Reporting of Observational Studies in Epidemiology (STROBE) checklist, which is appropriate for observational studies, to ensure transparency and reliability in reporting the findings. A total of 783 Japanese adults were recruited to form a large and demographically diverse cohort, representing the largest sample size to date for a nasal aesthetics study in this population. Three-dimensional imaging software (VECTRA, Vectra AI, Inc., San Jose, CA) was used to create standardized male and female nasal profiles, in which nasolabial and nasofacial angles were digitally manipulated with high precision (Figure 1). This approach minimized bias related to facial feature balance and enhanced the reproducibility and clinical relevance of the image-based evaluations.

### Study Population and Recruitment Process

A representative cohort of 783 Japanese adults was recruited from a range of professional backgrounds, including clinical staff and office workers, to capture a broad spectrum of aesthetic preferences. Participants were selected using stratified random sampling to ensure demographic diversity in terms of age, sex, and occupation. Specifically, recruitment was conducted through 2 primary sources: (1) aesthetic medical staff affiliated with Tokyo Chuo Beauty Clinic (Tokyo Chuo Biyougeka), and (2) office workers and general consumers who responded to a survey commissioned by Medical Frontier Inc., a general marketing company. This dual recruitment strategy facilitated the inclusion of both professionals in cosmetic medicine and members of the general public, thereby enhancing the diversity and relevance of the sample. Randomization was facilitated through internal communication, and participation was voluntary. To account for potential response bias, each participant was assigned a unique identifier, and the data were anonymized upon collection.

Participants were recruited through a combination of in-person invitations at affiliated cosmetic clinics and internal email communications within the medical group. The invitation outlined the purpose of the study and clarified that participation was entirely voluntary and unrelated to any ongoing treatments or consultations. No incentives were offered for participation.

### Image Generation and Presentation

**Figure 2.**
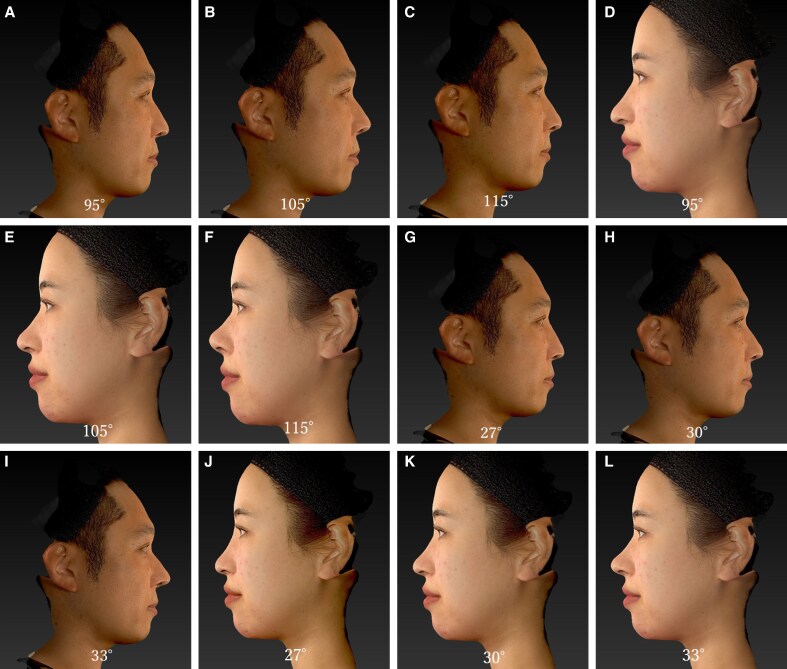
Simulated nasolabial angle variations. Male, 42 years old: no procedure performed. Female, 29 years old: no procedure performed. (A) Digitally modified male facial profile with a nasolabial angle of 95°. (B) Digitally modified male facial profile with a nasolabial angle of 105°. (C) Digitally modified male facial profile with a nasolabial angle of 115°. (D) Digitally modified female facial profile with a nasolabial angle of 95°. (E) Digitally modified female facial profile with a nasolabial angle of 105°. (F) Digitally modified female facial profile with a nasolabial angle of 115°. (G) Digitally modified male facial profile with a nasofacial angle of 27°. (H) Digitally modified male facial profile with a nasofacial angle of 30°. (I) Digitally modified male facial profile with a nasofacial angle of 33°. (J) Digitally modified female facial profile with a nasofacial angle of 27°. (K) Digitally modified female facial profile with a nasofacial angle of 30°. (L) Digitally modified female facial profile with a nasofacial angle of 33°.

Digitally modified nasal images were generated using the VECTRA 3D imaging system (Vectra AI, Inc.), which is known for its precision in facial analysis. The models used for image manipulation were Japanese adults who had never undergone any form of cosmetic or surgical procedures involving the nose or surrounding areas. Both individuals provided written informed consent for the use of their images in this research. We created 12 distinct nasal angle combinations representing variations in the nasolabial (95°, 105°, and 115°) and nasofacial angles (27°, 30°, and 33°), with each angle applied separately to standardized male and female facial models (Figure 2A, B). To ensure clarity and avoid interaction effects between variables, nasolabial and nasofacial angles were presented and evaluated independently rather than in combined configurations. The images depicted standardized male and female patients to ensure uniformity in the visual representation and facilitate accurate comparisons among participants.

The nasolabial angle was adjusted for Japanese individuals based on studies that traditionally defined the ideal range for women and men, incorporating the research by Takahashi and Naini et al assessed the nasofacial angle in 2016, establishing 30° as the standard with an acceptable range of 27° to 36°.^1,2^

### Survey Design and Data Collection

Participants completed a structured online survey administered through Google Forms (Google LLC, Alphabet Inc., Mountain View, CA), in which they assessed the attractiveness of each image by ranking them into 3 categories: “aesthetic,” “average,” and “not aesthetic.” The surveys were distributed through anonymous invitation links to ensure privacy, and the order of image presentation was randomized to minimize order effects. There were no specific inclusion criteria other than being a Japanese adult aged 18 years or older. To ensure data quality, we excluded any responses that were missing answers to key demographic variables, including age, sex, or occupation.

### Data Analysis

A normality test (Shapiro–Wilk) was performed to assess data distribution, and the Steel–Dwass test was used to evaluate statistically significant differences in aesthetic preferences across nasal angles. The sample size determination was based on previous studies, indicating an effect size of 0.3, achieving sufficient power (0.99) with a 5% significance level. The Academic Lounge academic consulting service oversaw the statistical analyses to ensure rigor in data interpretation and reporting. All statistical analyses were performed using JMP software (SAS Institute Inc., Cary, NC). *P*-values <.0001 were reported as “<0.0001” in accordance with the software's output limitations.

The weighted average was used to determine the overall aesthetic preference for the nasolabial and nasofacial angles in the male and female nasal profiles separately, as evaluated by all participants. Each rating category was assigned a numeric score: 3 points for “aesthetic,” 2 points for “average,” and 1 point for “not aesthetic.” The total score for each angle was calculated using the formula:


$$\begin{array}{rl} \mathrm{Total}\;\mathrm{score} & =(3\times \mathrm{number}\;\mathrm{of}\;"\mathrm{aesthetic}"\;\mathrm{votes})\\ & \;+(2\times \mathrm{number}\;\mathrm{of}\;"\mathrm{average}"\;\mathrm{votes})\\ & \;+(1\times \mathrm{number}\;\mathrm{of}\;"\mathrm{not}\;\mathrm{aesthetic}"\;\mathrm{votes}). \end{array}$$

The weighted average angle was then determined using the formula:


$$\mathrm{Weighted}\;\mathrm{average}\;\mathrm{angle}=\frac{\sum \;(\mathrm{Angle}\times \mathrm{Total}\;\mathrm{score})}{\sum \;\mathrm{Total}\;\mathrm{score}}.$$

### Ethical and Privacy Considerations

To safeguard participant confidentiality, we distributed anonymous survey links and stored the data securely in compliance with the institutional guidelines. Informed consent was obtained both verbally and in writing from all participants for participation and use of photographs for research purposes. All procedures were aligned with ethical standards to uphold participants' rights and data privacy. The study was conducted in compliance with ethical standards and approved by Tokyo Chuo Beauty Clinic (approval no. UMEDAERB-2024Jul001), and it adhered to the 1964 Declaration of Helsinki and its subsequent revisions, including the most recent amendment in Fortaleza, Brazil, in 2013.

## RESULTS

A total of 783 Japanese adults participated in this study, most of whom were women (88.5%). The largest age group was 25 to 29 years (43.3%). The detailed demographic information is presented in Table 1. Using the Steel–Dwass test, differences in aesthetic evaluations of preferred nasolabial and nasofacial angles were analyzed and rated as “aesthetic” (3 points), “average” (2 points), or “not aesthetic” (1 point).

**Table 1.** Demographic Information of Participants

Demographic category	*n*	(%)
Age, years		
18-24	149	19.0293742
25-29	321	40.9961686
30-34	173	22.0945083
35-39	77	9.8339719
>40	63	8.04597701
Sex		
Male	90	11.4942529
Female	693	88.5057471
Occupation		
Office worker	270	34.4827586
Nurse	127	16.2196679
Receptionist	112	14.3039591
Nursing assistant	108	13.7931034
Beauty counselor	97	12.3882503
Cosmetic surgeon	44	5.61941252
** Social networking service (**SNS) marketer	25	3.19284802

### Primary Outcomes: Preferred Nasolabial and Nasofacial Angles

#### Nasolabial Angle Preferences (Male)

**Figure 3.**
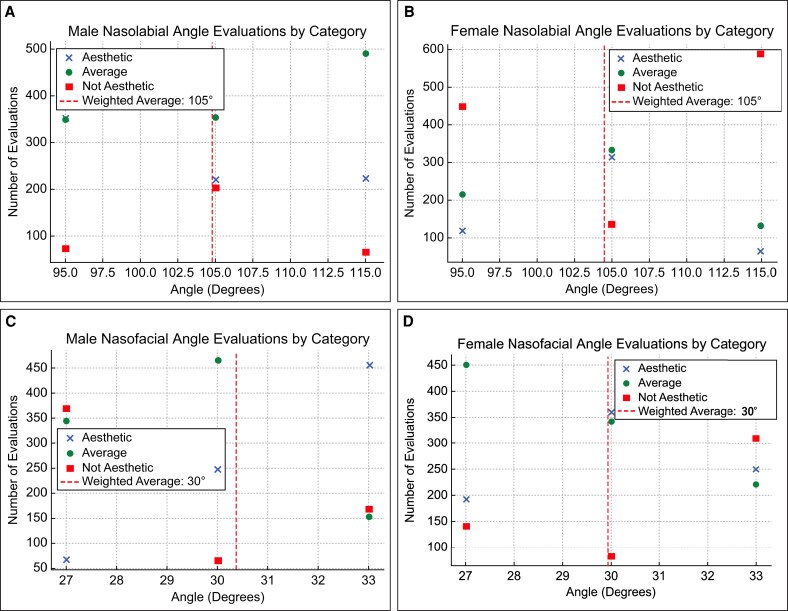
Ideal angles by weighted average. (A) Male nasolabial angle. (B) Female nasolabial angle. (C) Male nasofacial angle. (D) Female nasofacial angle.

Aesthetic evaluation revealed a significant preference for male nasolabial angles of 105° < 115° < 95° (all pairwise comparisons, *P* < .0001; note, values below .0001 are not reported with greater precision because of software limitations). The 95° angle was rated the highest, suggesting that it was the most aesthetically preferred angle for men (Figure 3A).

#### Nasolabial Angle Preferences (Female)

For female patients, 105° was rated as the most aesthetically pleasing, with a trend of 115° < 95° < 105° (all pairwise comparisons, *P* < .0001; Figure 3B).

#### Nasofacial Angle Preferences (Male)

Male participants rated 33° as the preferred nasofacial angle, with a trend of 27° < 30° < 33° (all pairwise comparisons, *P* < .0001; Figure 3C).

#### Nasofacial Angle Preferences (Female)

For females, the nasofacial angle of 30° was rated as the most aesthetic, followed by 33°, and then 27°. Pairwise comparisons revealed significant differences between 30° and 27° (*P* < .0001), 33° and 27° (*P* = .0006), and 30° and 33° (*P* < .0001; Figure 3D).

Based on these results, a clear difference was observed in the ideal nasal angles between men and women. The most aesthetically preferred angles were a 95° nasolabial angle and a 33° nasofacial angle for men and a 105° nasolabial angle and a 30° nasofacial angle for women.

Furthermore, when calculating the ideal angles using the weighted average, the ideal nasolabial angle for men was 105°, and the ideal nasofacial angle was 30°. For women, the ideal nasolabial and nasofacial angles were 105° and 30°, respectively (Figure 3).

### Secondary Outcomes: Sex, Age, and Occupational Analysis

#### Sex Differences in Angle Preferences

**Figure 4.**
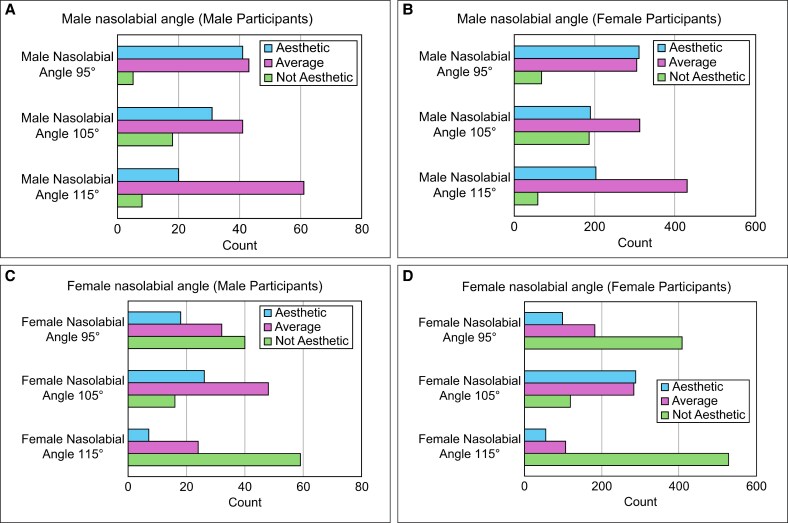

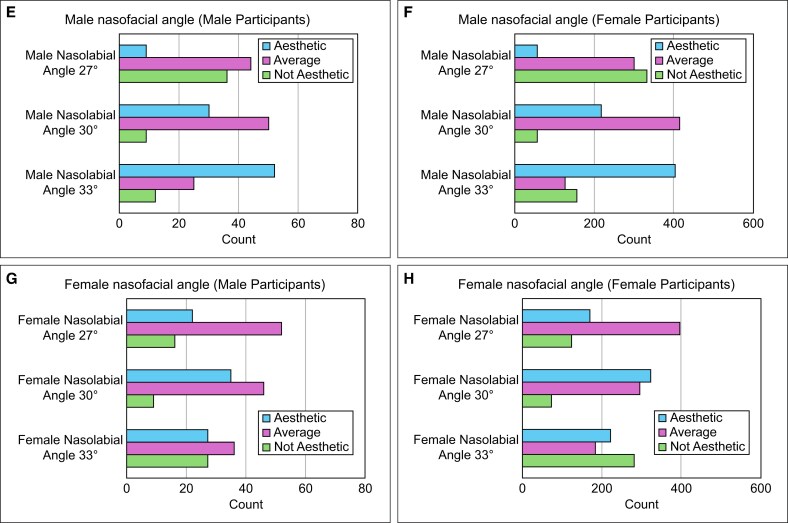
Nasolabial and nasofacial angle preferences based on sex. (A) Male nasolabial angle (male participants). (B) Male nasolabial angle (female participants). (C) Female nasolabial angle (male participants). (D) Female nasolabial angle (female participants). (E) Male nasofacial angle (male participants). (F) Male nasofacial angle (female participants). (G) Female nasofacial angle (male participants). (H) Female nasofacial angle (female participants).

Male and female participants showed similar trends in nasolabial and nasofacial preferences, with no statistically significant sex-based differences (Figure 4, Table 2).

**Table 2.** Gender Comparison in Nasolabial and Nasofacial Angle Preferences

Analysis type	Participants	In order of preference	Significant differences
Male nasolabial angle	Male	105° < 95°, 115° < 95°	95° > 105°, 115°
Male nasolabial angle	Female	105° < 115° < 95°	115° > 105°, 95° > 115°
Female nasolabial angle	Male	115° < 95° < 105°	95° > 115°, 105° > 95°
Female nasolabial angle	Female	115° < 95° < 105°	95° > 115°, 105° > 95°
Male nasofacial angle	Male	27° < 30° < 33°	30° > 27°, 33° > 30°
Male nasofacial angle	Female	27° < 30° < 33°	30° > 27°, 33° > 30°
Female nasofacial angle	Male	33° < 30°	30° > 33°, 27° = 30° = 33°
Female nasofacial angle	Female	33° < 27° < 30°	27° > 33°, 30° > 27°

#### Age Analysis

**Figure 5.**
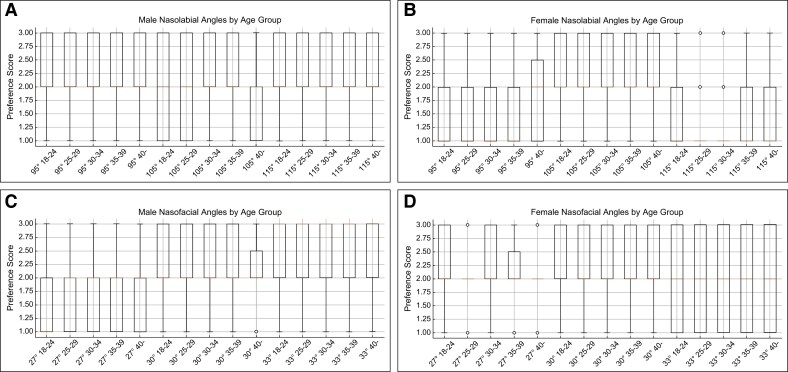
Age-based analysis of nasolabial and nasofacial angle preferences. (A) Male nasolabial angle. (B) Female nasolabial angle. (C) Male nasofacial angle. (D) Female nasofacial angle.

No significant differences were found in the preferred angles across age groups for either the nasolabial or nasofacial angles (Figure 5).

#### Occupational Differences

Occupational analysis showed significant differences in female nasolabial angles at 105° and 115° (*P* < .0001), particularly between office workers and those in other occupations, with nursing assistants and receptionists showing higher preferences for these angles (Table 3).

**Table 3.** Occupational Analysis of Nasolabial Angle Preferences (Significant Differences)

Kruskal–Wallis test		
Angle	*χ* ^2^	*P*-value
Male nasolabial angle—95°	6.78	.342
Male nasolabial angle—105°	12	.062
Male nasolabial angle—115°	4.2	.649
Female nasolabial angle—95°	11.53	.073
Female nasolabial angle—105°	18.98	.004^a^
Female nasolabial angle—115°	29.88	<.001^a^
Male nasofacial angle—27°	9.87	.13
Male nasofacial angle—30°	7.29	.295
Male nasofacial angle—33°	9.3	.157
Female nasofacial angle—27°	7.38	.287
Female nasofacial angle—30°	13.02	.043^a^
Female nasofacial angle—33°	11.34	.078

Post hoc pairwise comparisons with Bonferroni correction		
Angle	Pairwise comparison	*P*-value
Female nasolabial angle—105°	Office worker vs receptionist	.0325
Female nasolabial angle—105°	Office worker vs nursing assistant	.0287
Female nasolabial angle—115°	Office worker vs beauty counselor	.00023
Female nasolabial angle—115°	Office worker vs nursing assistant	.0277

Occupation	Male nasolabial angle preferences	Male nasofacial angle preferences	Female nasolabial angle preferences	Female nasofacial angle preferences
Office worker	115° < 95°, 105° < 95°	27° < 30°, 27° < 33°	95° < 105°, 115° < 105°	27° < 30°, 33° < 30°
Nurse	105° < 95°	27° < 30°, 27° < 33°	115° < 95° < 105°	33° < 30°
Receptionist	115° < 95°, 105° < 95°	27° < 30° < 33°	115° < 95° < 105°	27° < 30°, 33° < 30°
Nursing assistant	105° < 115°, 105° < 95°	27° < 30°, 27° < 33°	115° < 95° < 105°	33° < 27° < 30°
Beauty counselor	105° < 115° < 95°	27° < 30° < 33°	115° < 95° < 105°	27° < 30°, 33° < 30°
Cosmetic surgeon	No significant difference	27° < 30° < 33°	115° < 95°, 115° < 105°	33° < 30°
SNS marketer	No significant difference	27° < 30°, 27° < 33°	95° < 105°, 115° < 105°	27° < 30°, 33° < 30°

^a^Indicates statisticallly significant differences (*P* < .05).

## DISCUSSION

In this study, we aimed to investigate the aesthetic preferences for nasolabial and nasofacial angles among a sample of 783 Japanese adults by examining differences based on sex, occupation, and age. In previous studies, the authors have documented the variability in ideal nasal aesthetics across different populations, with Caucasian groups generally favoring more obtuse nasolabial angles and Asian populations, including Japanese, typically preferring sharper angles.^3,4^ However, existing studies lack large-scale data specifically for Japanese adults, limiting the applicability of their findings. By addressing this gap, our study offers novel insights into current aesthetic preferences in Japan, which are valuable for both domestic and international practitioners treating East Asian patients.

Our findings demonstrated clear sex-specific preferences: men rated a nasolabial angle of 95° and a nasofacial angle of 33° as the most aesthetic, whereas women preferred a nasolabial angle of 105° and a nasofacial angle of 30°. Although the weighted average indicated that 105° was the most preferred nasolabial angle among male profiles, a closer inspection of the distribution reveals that this angle also received the highest number of “not aesthetic” ratings and the lowest number of “aesthetic” ratings. This suggests a polarized response pattern, where some participants rated it highly, whereas others found it unattractive. Therefore, the use of a weighted average, although statistically valid, may not fully capture the subjective distribution of aesthetic preferences. Future studies may benefit from incorporating additional distributional metrics, such as modal values or interquartile ranges, to provide a more nuanced interpretation of preference trends. Occupational differences further revealed that certain angles, such as the nasolabial angle of 105°, were rated more highly by professionals with frequent social interactions, such as receptionists and beauty counselors. Notably, age did not significantly influence nasal angle preferences, suggesting a stable aesthetic perception across age groups in Japan. This aligns with previous studies reporting consistent aesthetic preferences for other facial features regardless of age, indicating that nasal aesthetic evaluations may remain stable among Japanese adults, irrespective of age.^5^

The authors of previous studies have reported the general preferences for nasal angles in Asian populations. Takahashi estimated the ideal nasolabial angle for Japanese adults to be ∼118.32°, based on small samples and specific demographic groups.^1^ However, previous research has traditionally defined ideal nasolabial angles in the range of 95° to 100° for men and 103° to 108° for women, with some studies suggesting no significant changes in these values in recent years.^6,7^ Other studies have indicated that the ideal angles for men and women become closer, showing reduced variation by sex.^8-10^ However, our results indicate a shift toward sharper angles, with the Japanese participants in our study favoring a 95° nasolabial angle for men and 105° for women. This trend may reflect an evolution in aesthetic ideals, potentially influenced by global beauty trends and the increasing impact of social media on Japanese beauty standards.^11,12^

Additionally, the authors of previous studies on nasofacial angles, such as Naini et al, proposed a standard of ∼30° as ideal, with a range of 27° to 36° considered acceptable.^2^ Our findings generally align with this range but also underscore slight sex-specific variations that may hold clinical significance in rhinoplasty for Japanese patients.

A significant strength of this study is its large and large sample size, which enhances the generalizability of our findings. Previous studies with a limited number of participants were unable to provide definitive aesthetic standards for the Japanese population. Furthermore, the application of advanced 3D imaging technology (VECTRA) allowed us to precisely control and present the nasal angles, providing consistent visual representations that facilitated accurate aesthetic evaluation. Clinically, our results emphasize the need for customized surgical approaches that account for demographic factors such as sex and occupation, particularly when adjusting for nasal angles in rhinoplasty. Tailored approaches in rhinoplasty can enhance patient confidence and satisfaction, ultimately contributing to a positive perception of surgical outcomes.^11,13^

Although this study provides valuable data, some limitations should be acknowledged. First, the majority of participants were women (88.5%) and between 18 and 40 years old, which may limit the broader applicability of our results. However, these demographics align with the profile of patients most likely to seek nasal cosmetic surgery, lending relevance to our findings.^14,15^ Second, this study was conducted in a single-center, urban Japanese clinical setting, and the participants were primarily recruited from within the healthcare field. This sampling approach likely constitutes a convenience sample, which may introduce selection bias and limit the generalizability of the findings. Although the sample reflects a population with a high likelihood of undergoing cosmetic procedures, it does not fully represent the broader Japanese population, especially older individuals and males from other occupational backgrounds. Third, the use of 1 male and 1 female images for each angle adjustment may have limited participants' evaluations, because the overall facial balance can unconsciously influence nasal aesthetics. Fourth, although angle adjustments were performed using standardized 3D imaging software (VECTRA), minor unintended changes occurred in surrounding anatomical structures. For example, in the male profile with a nasolabial angle of 115°, the upper lip inclination appeared more vertical compared with other conditions, which may have reduced the perceived degree of tip rotation. Similarly, in the male nasofacial angle series, the position of the sellion (Se) point varied, resulting in changes in nasal length (Se–Pn distance). In contrast, the female model maintained greater consistency in these adjacent features. Consequently, participant judgments may have been influenced by co-occurring changes beyond the targeted angles, particularly in the male images. These technical limitations reflect current challenges in achieving perfect anatomical isolation using automated facial modeling systems. Future studies should expand these findings by including a broader demographic range, particularly incorporating Southeast Asian, Central Asian, and Caucasian populations and a multicenter approach, as this would provide a more comprehensive understanding of aesthetic preferences across different Japanese populations. Additionally, research exploring generational and cross-cultural shifts in nasal aesthetic preferences could provide valuable insights for establishing more universally applicable standards as societal influences, such as social media and globalization, increasingly shape beauty norms.^16^ The use of online crowd-sourcing survey platforms may also help obtain a more demographically balanced and representative sample, thereby improving the external validity of future research. Incorporating additional angles, such as the nasofrontal angle, could further enhance the scope of facial analysis, offering a more holistic perspective on nasal aesthetics. Furthermore, although we assessed nasolabial and nasofacial angles independently in this study, future research should examine the aesthetic implications of combined angle configurations (eg, specific nasolabial–nasofacial pairings). Investigating potential interaction effects among multiple nasal angles could provide deeper insights into how overall nasal harmony shapes aesthetic perception. This study provides foundational data that may inform more personalized and demographically tailored approaches to rhinoplasty in Japanese patients. These findings highlight the importance of culturally sensitive, demographically informed rhinoplasty strategies that honor individual identity while aligning with evolving global beauty standards.^17^

## CONCLUSIONS

This study effectively identified sex- and occupation-specific preferences for nasolabial and nasofacial angles among a representative sample of Japanese adults likely to pursue cosmetic surgery. Specifically, a nasolabial angle of 95° and nasofacial angle of 33° were rated as most aesthetic in male nasal profiles, whereas a nasolabial angle of 105° and nasofacial angle of 30° were preferred in female nasal profiles. These findings provide foundational insights for developing personalized rhinoplasty strategies that consider demographic factors, particularly sex and the degree of social interaction. Because cosmetic surgery continues to globalize and evolve in response to shifting cultural norms, the adoption of a nuanced and culturally sensitive approach becomes increasingly important. Recognizing patient-specific aesthetic preferences not only improves surgical outcomes but also affirms individual identity and enhances overall patient satisfaction.
